# Supplementary material for: ProteinShader: illustrative rendering of macromolecules
Source: BMC Struct Biol. 2009 Mar 30;9:19. doi: 10.1186/1472-6807-9-19 (PMC2672931; doi:10.1186/1472-6807-9-19)
Supplement: Additional file 1 — ProteinShader program without source code. This compressed file contains the complete ProteinShader program including associated libraries, but no source code. A README.txt file gives an overview of the ProteinShader distribution, and the index.html file in the help subdirectory has directions on getting started with the program as well as a set of tutorials. [file 1472-6807-9-19-S1.zip › ProteinShader-beta-0_9_4-binary/help/api/org/proteinshader/structure/visitor/demos/ModifierVisitorDemo.html]

ModifierVisitorDemo (ProteinShader API)


|  |  |  |
| --- | --- | --- |
| **Overview** \| **Package** \| **Class** \| **Use** \| **Tree** \| **Deprecated** \| **Index** \| **Help** | |  |
| PREV CLASS   **NEXT CLASS** | **FRAMES**    **NO FRAMES**     **All Classes** |  |
| SUMMARY: NESTED \| FIELD \| CONSTR \| METHOD | DETAIL: FIELD \| CONSTR \| METHOD |  |


---


## org.proteinshader.structure.visitor.demos Class ModifierVisitorDemo

```
java.lang.Object
  org.proteinshader.structure.visitor.demos.ModifierVisitorDemo
```

---

``` public class ModifierVisitorDemo extends Object ```

Temporary class for testing the ModifierVisitor class.
USAGE:
java -cp . path/ModifierVisitorDemo inputFilename outputFilename
where
path = edu/harvard/fas/jrweber/molecular/structure/visitor/demos/
After reading 'inputFilename' and creating a Structure, the
Structure will be written as an object dump to a file named
'outputFilename.out'.
The Helices of the Structure are modified by changing their shape to
CYLINDER, their visiblity to OPAQUE, and setting all of their Atoms
and Bonds to INVISIBLE. Modifications will then be made and written
to 'outputFilename-Modified.out".

---

| **Field Summary** | |
| --- | --- |
| `static String` | `PATH` |
| `static String` | `usageMessage` |


**Constructor Summary**

`ModifierVisitorDemo()`
  
          Constructs a PDBStructureReaderDemo.


| **Method Summary** | |
| --- | --- |
| `String` | `getFilenameOrDie(String[] cmdLineArgs, int index)`             Returns the requested command line argument if it is present. |
| `static void` | `main(String[] args)`             Uses a PDBStructureReader to read a PDB file and create a Structure, and then uses a WriterVisitor to write the contents of the Structure to an output file. |
| `void` | `modify(Structure structure)`             The Helices of the Structure are modified by changing their shape to CYLINDER and setting all of their Atoms and Bonds to INVISIBLE. |
| `void` | `printBadLines(List<String> badLines)`             Prints the bad lines from the PDB file to standard out. |
| `void` | `writeStructure(Structure structure, String filename)`             Writes a Structure to a file by using a SFWriterVisitor. |

**Methods inherited from class java.lang.Object**

`clone, equals, finalize, getClass, hashCode, notify, notifyAll, toString, wait, wait, wait`

**Field Detail**

### PATH

```
public static final String PATH
```

**See Also:**: Constant Field Values

---


### usageMessage

```
public static final String usageMessage
```

**See Also:**: Constant Field Values


**Constructor Detail**

### ModifierVisitorDemo

```
public ModifierVisitorDemo()
```

:   Constructs a PDBStructureReaderDemo.


**Method Detail**

### writeStructure

```
public void writeStructure(Structure structure,
                           String filename)
                    throws VisitorException
```

:   Writes a Structure to a file by using a SFWriterVisitor. If
    filename does not exist, it will be created. If it already
    exists, it will be overridden.

    :   **Parameters:**: `structure` - the Structure to write.: `filename` - the name of the file to write to. **Throws:**: `VisitorException` - if an IO error occurs.

---


### modify

```
public void modify(Structure structure)
            throws VisitorException
```

:   The Helices of the Structure are modified by changing their shape
    to CYLINDER and setting all of their Atoms and Bonds to INVISIBLE.

    :   **Parameters:**: `structure` - the Structure to Modify. **Throws:**: `VisitorException` - if an error occurs while modifying the Structure.

---


### printBadLines

```
public void printBadLines(List<String> badLines)
```

:   Prints the bad lines from the PDB file to standard out. To save
    bad lines to a file, use the redirection command for standard out,
    ' > filename'.

    :   **Parameters:**: `badLines` - the bad lines to print.

---


### getFilenameOrDie

```
public String getFilenameOrDie(String[] cmdLineArgs,
                               int index)
```

:   Returns the requested command line argument if it is present.
    Otherwise, prints a usage message to standard out and then
    terminates the program.

    :   **Parameters:**: `cmdLineArgs` - the command line arguments.: `index` - the index position to get the filename from.

---


### main

```
public static void main(String[] args)
```

:   Uses a PDBStructureReader to read a PDB file and create a
    Structure, and then uses a WriterVisitor to write the contents
    of the Structure to an output file.


---


|  |  |  |
| --- | --- | --- |
| **Overview** \| **Package** \| **Class** \| **Use** \| **Tree** \| **Deprecated** \| **Index** \| **Help** | |  |
| PREV CLASS   **NEXT CLASS** | **FRAMES**    **NO FRAMES**     **All Classes** |  |
| SUMMARY: NESTED \| FIELD \| CONSTR \| METHOD | DETAIL: FIELD \| CONSTR \| METHOD |  |


---

# *Copyright © 2007-2008*
